# Fluorescence-Guided Thoracoscopic Surgery Using Indocyanine Green (ICG) in Canine Cadavers: A Descriptive Evaluation of Video-Assisted (VATS) and Robot-Assisted (RATS) Approaches

**DOI:** 10.3390/ani15243519

**Published:** 2025-12-05

**Authors:** Francisco M. Sánchez-Margallo, Lucía Salazar-Carrasco, Manuel J. Pérez-Salazar, Juan A. Sánchez-Margallo

**Affiliations:** 1 Scientific Direction, Jesús Usón Minimally Invasive Surgery Center, 10171 Cáceres, Spain; 2 Bioengineering and Health Technologies Unit, Jesús Usón Minimally Invasive Surgery Center, 10171 Cáceres, Spain; lsalazar@ccmijesususon.com (L.S.-C.); mjperez@ccmijesususon.com (M.J.P.-S.); jasanchez@ccmijesususon.com (J.A.S.-M.)

**Keywords:** chylothorax, canine, thoracic duct, indocyanine green, near-infrared fluorescence, video-assisted thoracoscopy, robotic-assisted surgery

## Abstract

Precise intraoperative identification of the canine thoracic duct remains challenging due to anatomical variability and limited visualization. This exploratory cadaveric feasibility study aimed to describe the technical applicability of fluorescence-guided thoracic duct mapping using video-assisted thoracoscopy (VATS) and robot-assisted thoracoscopy (Versius™ system). Four adult Beagle cadavers underwent bilateral thoracoscopic exploration after intranodal injection of indocyanine green (ICG, Verdye^®^, 0.05 mg/kg; 0.5 mL). Near-infrared (NIR) fluorescence imaging enabled real-time visualization of the thoracic duct and its branches. Fluorescence quality was quantitatively characterized using signal-to-noise ratio (SNR), contrast-to-noise ratio (CNR), and contrast resolution (CR) calculated from standardized image frames. Both approaches achieved successful duct identification in all cadavers. VATS provided brighter overall fluorescence, whereas the robotic-assisted approach offered stable imaging, enhanced instrument dexterity, and improved duct-to-background discrimination. These findings confirm the feasibility of fluorescence-guided thoracic duct identification using both minimally invasive modalities in canine cadavers. The standardized assessment of optical parameters proposed here may support future in vivo studies to optimize imaging protocols and evaluate the clinical impact of fluorescence-guided thoracic duct surgery in dogs.

## Simple Summary

Chylothorax in dogs often requires surgery to ligate the thoracic duct, but anatomical variation and poor visualization can lead to incomplete ligation and recurrence. We tested two minimally invasive approaches: video-assisted thoracoscopy (VATS) and robot-assisted thoracoscopy with the Versius™ platform (RATS) to visualize the duct using near-infrared (NIR) fluorescence after indocyanine green (ICG) injection in four canine cadavers. Both methods identified the duct in all specimens. VATS produced brighter overall fluorescence, whereas RATS delivered higher duct-to-background contrast and greater precision due to three-dimensional imaging and wristed instruments. These findings support the use of fluorescence-guided minimally invasive surgery as a practical approach to enhance thoracic duct mapping. In vivo studies are necessary to confirm whether the robot-assisted approach provides superior outcomes and reduced recurrence rates.

## 1. Introduction

Chylothorax is a rare yet clinically significant disorder in canines, characterized by the accumulation of chyle within the pleural cavity. Untreated, it leads to respiratory failure, weight loss, and progressive cachexia [1,2]. The etiology of chylothorax encompasses trauma, neoplasia, heart disease, and congenital anomalies. Notably, many cases remain idiopathic, presenting treatment challenges [3,4]. Idiopathic chylothorax presents difficulties due to the anatomical variability of the thoracic duct and cisterna chyli, potentially including collateral channels or plexiform networks that are not systematically documented in standard anatomical references [3,5]. These anatomical variations substantially increase the risk of incomplete ligation and, consequently, lead to persistence of effusion [6,7].

Thoracic duct ligation (TDL) remains the cornerstone of surgical treatment. It can be performed in conjunction with complementary procedures, such as cisterna chyli ablation (CCA) or subtotal pericardiectomy, which have been associated with improved long-term outcomes [8,9,10]. Minimally invasive thoracoscopic surgery (VATS) has demonstrated reduced postoperative morbidity and enhanced visualization compared to open thoracotomy, although both approaches are still employed in clinical practice [8,9]. The success rates of thoracoscopic TDL range from 80% to 95%, particularly when combined with advanced techniques [10,11].

Accurate preoperative imaging and intraoperative imaging have significantly improved surgical outcomes. In the preoperative phase, computed tomography lymphangiography (CTLG) has emerged as a pivotal imaging modality for the detection of the thoracic duct in canine patients. It offers comprehensive high-resolution three-dimensional mapping of the lymphatic anatomy. Techniques such as injection into the mesenteric lymph nodes [12,13], contrast administration into the popliteal lymph nodes [14], and subcutaneous or intrametatarsal injections [15,16] have been validated and demonstrate their efficacy in characterizing anatomical variability and collateral branches in idiopathic chylothorax [17]. During surgery, near-infrared (NIR) indocyanine green (ICG) fluorescence imaging has emerged as a promising real-time tool for lymphatic visualization and mapping in both experimental and clinical veterinary contexts [6,18]. However, its use remains limited in routine referral practice, and further standardization of dose, injection technique, and imaging parameters is required. Developing reproducible cadaveric models represents an essential step toward refining these protocols and evaluating their feasibility prior to clinical application.

Robotic-assisted thoracoscopic surgery (RATS) is gaining interest in veterinary medicine, drawing inspiration from its successful implementation in human thoracic surgery. Robotic systems provide three-dimensional visualization, tremor reduction, and articulated instruments to enhance precision [19,20,21]. Nevertheless, their adoption is hindered by cost, accessibility, and the requisite training. The Versius™ robotic platform (CMR Surgical; Cambridge, UK), characterized by its modular design and fluorescence capabilities, presents a suitable modality for assessing these advantages in veterinary thoracic procedures.

This exploratory cadaveric feasibility study was designed to describe the technical applicability of fluorescence-guided thoracic duct identification using both video-assisted (VATS) and robot-assisted (Versius™) thoracoscopic approaches. Rather than establishing a comparative analysis, the study aimed to demonstrate feasibility and characterize the qualitative and quantitative imaging parameters of ICG–NIR fluorescence under standardized conditions. These findings are intended to guide the design of future in vivo studies on the surgical management of canine idiopathic chylothorax.

## 2. Materials and Methods

### 2.1. Cadaveric Specimens

Four adult Beagle cadavers (two males and two females; body weight: 13–17 kg) were used in this study. A cadaveric model was selected to reproduce realistic anatomical topography, spatial constraints, and light reflection within the thoracic cavity, which cannot be replicated in vitro. The cadavers were obtained from animals euthanized for reasons unrelated to this research and stored at −20 °C until use. To preserve tissue integrity and maintain optical properties like in vivo conditions, all procedures were performed within 24 h after complete thawing. Cadavers were handled according to institutional and national guidelines for the use of animal remains in research and teaching activities.

### 2.2. Study Design

This exploratory cadaveric feasibility study was designed to describe and characterize fluorescence-guided thoracic duct identification using two minimally invasive approaches (VATS and RATS), under standardized conditions, without inferential comparison between modalities.

Each cadaver underwent both thoracoscopic approaches sequentially, alternating the side of access (left or right hemithorax) between specimens to reduce anatomical and procedural bias.

The primary endpoint was satisfactory visualization of the TD and its branches using near-infrared (NIR) fluorescence after injection of indocyanine green (ICG). Secondary endpoints included the time to initial fluorescence, contrast-to-noise ratio (CNR), and contrast resolution (CR).

### 2.3. Indocyanine Green Protocol

A ventral midline celiotomy was performed to expose the jejunal lymphatic chain. A mesenteric lymph node (3–5 mm in diameter) in the proximal jejunum was identified and injected with 0.5 mL of indocyanine green (ICG) solution (0.05 mg/kg; Verdye^®^, Diagnostic Green GmbH, Aschheim-Dornach, Germany) diluted 1:1 with sterile saline using a 25G needle. Gentle manual compression was applied for 30 s after injection to minimize leakage and promote lymphatic absorption.

After injection, a waiting period of approximately 10 min was observed before thoracoscopic exploration to allow adequate lymphatic transport and ductal filling.

### 2.4. Thoracoscopic Approaches

#### 2.4.1. Video-Assisted Thoracoscopic Surgery (VATS)

**Figure 1:**
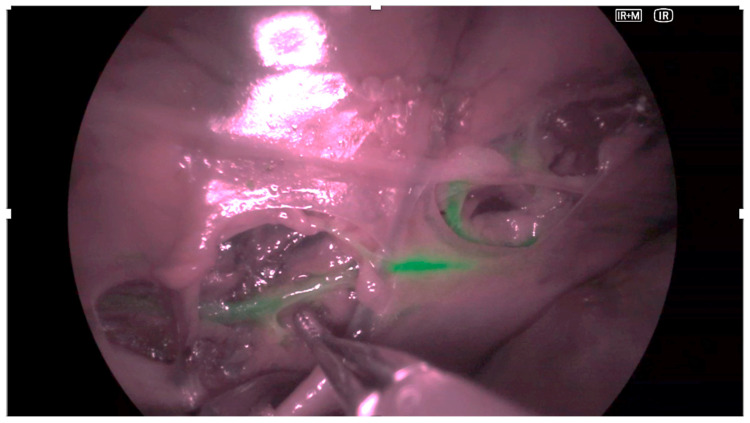
Representative intraoperative image obtained with video-assisted thoracoscopic surgery (VATS) using the VISERA ELITE III imaging system in a cadaveric model. The thoracic duct (TD) is highlighted by near-infrared (NIR) fluorescence after intranodal indocyanine green (ICG) injection. The image illustrates the feasibility of fluorescence-guided duct identification under cadaveric conditions.

VATS was performed using the Olympus VISERA ELITE III imaging system (Olympus Medical Systems; Tokyo, Japan). A three-port approach was used. A 5 mm, 30° thoracoscope was inserted through the 7th intercostal space (ICS), with two additional 5 mm working ports placed in the 5th and 9th ICS. The thoracic duct was located next to the aorta, and its course was traced. Fluorescence was visualized using the integrated NIR mode of the VISERA III system (Figure 1).

Fluorescence intensity, duct delineation, and background contrast were qualitatively assessed by two independent observers during each 5 min inspection interval.

#### 2.4.2. Robot-Assisted Thoracoscopic Surgery (RATS)

**Figure 2:**
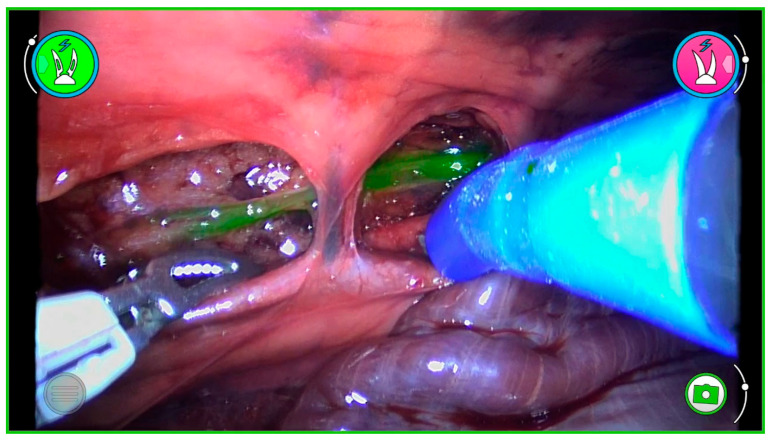
Representative intraoperative image obtained with robotic-assisted thoracoscopic surgery (RATS) using the Versius™ system in a cadaveric model. The thoracic duct (TD) is visualized by near-infrared fluorescence with the greenICG™ module, showing a distinct contrast pattern against mediastinal structures. The figure demonstrates the technical capability of robotic fluorescence imaging for lymphatic mapping.

RATS was performed using the Versius™ robotic system (CMR Surgical; Cambridge, UK). A three-arm setup was employed: the robotic camera (with the greenICG™ fluorescence module; CMR Surgical) was positioned at the 7th ICS, while two robotic arms were inserted at the 5th and 9th ICS. The system provided high-definition three-dimensional images and wristed instruments for dissection (Figure 2).

Instrument docking, camera stability, and operator ergonomics were documented throughout each procedure using predefined observational criteria. The operator ergonomics was systematically evaluated according to three criteria: surgeon posture and comfort, instrument handling and precision, and camera control and stability. An independent observer documented these aspects throughout each procedure for both VATS and RATS.

Both methods followed the same inspection protocol, with systematic exploration of both hemithoraces every 5 min for up to 60 min after ICG injection. The thoracic duct was identified by its distinctive linear fluorescence pattern adjacent to the aorta. Once located, the ducts were dissected and ligated using endoscopic clips or sutures.

No quantitative comparison of fluorescence signal was conducted between techniques; all observations were analyzed descriptively to characterize technical feasibility and visualization quality.

### 2.5. Image Acquisition and Analysis

**Figure 3:**
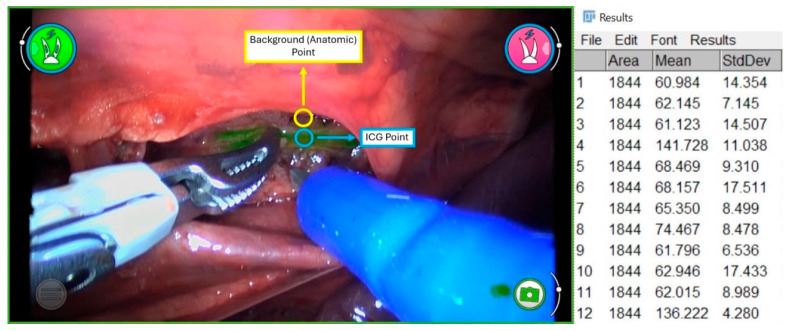
Example of quantitative frame analysis using Fiji software (ImageJ, NIH, Bethesda, MD, USA). Analysis performed on representative frames (*n* = 25 per specimen). Twelve regions of interest (ROIs) were selected per frame, six within the fluorescent thoracic duct and six in adjacent background tissue. Mean pixel intensity and standard deviation were extracted to calculate the signal-to-noise ratio (SNR), contrast-to-noise ratio (CNR), and contrast resolution (CR). Each ROI encompassed an area of 1844 pixels.

All procedures were documented and recorded on video for subsequent quantitative analysis. Twenty-five representative frames were randomly selected from each case. Image analysis was conducted using Fiji software (v1.54f, ImageJ; NIH, Bethesda, MD, USA) (Figure 3). In this regard, regions of interest (ROIs) were delineated in fluorescent segments of the thoracic duct (foreground) and surrounding tissue (background). Each ROI covered approximately 1844 pixels. Six ROIs were selected from duct areas and six from adjacent tissue per frame. To account for side-dependent anatomical variation, image analysis was standardized by defining ROIs according to fluorescence pattern and relative position to the aorta, regardless of side.

The following quantitative parameters were calculated as surrogate indicators of image quality [22]:

Signal-to-Noise Ratio (SNR): the mean duct intensity divided by the standard deviation of the background intensity.

Contrast-to-Noise Ratio (CNR): (mean duct intensity—mean background intensity) divided by the standard deviation of the background.

Contrast Resolution (CR): (CNR ÷ mean background intensity) × 100%.

All measurements were performed by two expert independent observers using identical light intensity, working distance, and camera settings across both systems. These parameters describe the visibility of fluorescence and its clinical relevance in improving duct identification under standardized optical conditions.

### 2.6. Statistical Analysis

Statistical analysis was performed using Jamovi software (version 2.3, The Jamovi Project, Sydney, Australia). Normality was assessed with the Shapiro–Wilk test. Given the exploratory and descriptive nature of this cadaveric feasibility study, statistical tests were used only to summarize variability rather than to establish inferential comparisons between platforms. Results are expressed as mean ± standard deviation (SD) or median (range), as appropriate.

## 3. Results

### 3.1. Thoracic Duct Visualization

Near-infrared (NIR) fluorescence mediated by ICG successfully identified the thoracic duct in all cadavers (*n* = 4). The mean time to initial visualization was 24.3 ± 9.7 min (range: 17–38 min). No differences were observed between VATS and RATS in the time to first detection of the TD. In one cadaver, a second ICG bolus was required to achieve adequate fluorescence intensity, after which visualization was obtained. This variability likely reflected differences in lymphatic filling or ICG diffusion rather than imaging performance. All procedures allowed reliable duct recognition, confirming the feasibility of both approaches in a cadaveric setting (Table 1).

### 3.2. Operative Times

The mean operative time (from incision to closure) was 35.0 ± 4.5 min for VATS and 38.2 ± 5.1 min for RATS. The robotic system required a slightly longer setup and docking time. Given the cadaveric setting, operative time mainly reflected ergonomics and workflow rather than physiological factors.

### 3.3. Image Quality Metrics

Quantitative analysis of the 25 representative frames per cadaver revealed distinct optical profiles for both imaging systems (Table 2).

**Figure 4:**
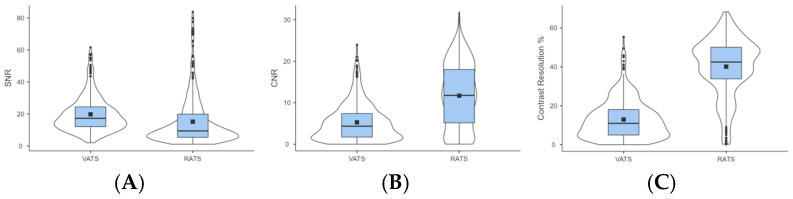
Descriptive comparison of fluorescence imaging parameters between VATS and RATS in canine cadavers. Boxplots illustrate the distribution of (**A**) signal-to-noise ratio (SNR); (**B**) contrast-to-noise ratio (CNR); and (**C**) contrast resolution (CR). Values are expressed as mean ± standard deviation (SD). No inferential statistics were applied due to the exploratory nature of the study.

VATS showed a higher signal-to-noise ratio (SNR) than RATS (19.7 ± 10.9 vs. 15.2 ± 15.0) (Figure 4A). RATS achieved a higher contrast-to-noise ratio (CNR) (11.7 ± 7.6 vs. 5.3 ± 4.4) (Figure 4B) and contrast resolution (CR) (40.1% ± 14.8% vs. 12.9% ± 9.8%) (Figure 4C).

These optical differences should be interpreted with caution, as they likely reflect intrinsic variations between imaging architectures rather than procedural performance. The Versius™ platform employs a stereoscopic 3D endoscope with dedicated optical filters and gain algorithms, while the Olympus VISERA ELITE III provides a 2D NIR channel with broader emission sensitivity. Consequently, SNR, CNR, and CR values primarily characterize the imaging systems rather than surgical dexterity or user performance, without direct clinical implications.

In practical terms, VATS produced brighter fluorescence overall, while RATS generated darker but higher-contrast images, facilitating precise identification of ductal structures. The cadaveric model allowed consistent imaging conditions without motion artifacts or hemodynamic interference, though it does not replicate in vivo lymphatic flow or perfusion.

### 3.4. Subjective Observations

The three-dimensional images and articulated instruments of the robotic system facilitated dissection and manipulation in confined thoracic spaces. The operator reported enhanced precision and stability with RATS, especially in complex anatomical regions, despite the images appearing dimmer compared to VATS. Fluorescence from adjacent vascular structures occasionally appeared but did not hinder duct identification. In one cadaver, fluorescence contrast decreased over time, likely due to dye redistribution rather than imaging limitations.

### 3.5. Limitations

Given that both imaging platforms differ in optical design, sensor calibration, and light-source intensity, direct quantitative comparison should be considered descriptive rather than competitive. The obtained parameters provide a technical characterization of image quality under standardized conditions but may vary in live animals due to dynamic lymphatic flow and perfusion. These results therefore demonstrate the feasibility and reproducibility of NIR-guided thoracic duct visualization, supporting future in vivo validation.

## 4. Discussion

This descriptive cadaveric study evaluated the feasibility of thoracic duct visualization using near-infrared (NIR) fluorescence with indocyanine green (ICG) during video-assisted (VATS) and robotic-assisted (RATS) thoracoscopic procedures in dogs. Both methods enabled reliable identification of the thoracic duct in all cases performed, demonstrating that ICG–NIR fluorescence can serve as a reliable tool for real-time lymphatic mapping in the thoracic cavity. The notable disparity observed in RATS is primarily attributed to the unique characteristics and configuration of the imaging system optics, including the 3D camera and its specialized fluorescence filters, rather than variations in surgical procedures. These optical advantages are inherent in the imaging system’s architecture and should not be interpreted as indicating superior surgical performance. Optical parameters exhibit variations that reflect the manufacturer’s distinct hardware and software design. It is important to note that image quality retains a subjective component, even when analyzed quantitatively. The study also highlighted some technical and ergonomic distinctions between the systems, which may influence visualization quality and workflow efficiency in future clinical applications.

Our results align with previous clinical and experimental evidence highlighting the benefits of ICG-NIR fluorescence in canine chylothorax surgery [4,6,18]. Steffey and Mayhew (2018) demonstrated the use of ICG fluorescence to identify all branches of the thoracic duct intraoperatively in canine clinical cases [6]. Similarly, Korpita et al. (2022) reported the reliable visualization of the duct within minutes of hepatic ICG injection in an experimental model [18]. In our case, ICG enabled real-time visualization of both small and accessory lymphatic branches. However, not all minor branches can be consistently detected due to the dynamic nature of lymphatic flow.

In this cadaveric setting, differences in image metrics such as signal-to-noise and contrast ratios reflected the intrinsic optical and sensor characteristics of the two imaging systems rather than true performance disparities. The robotic system provided greater contrast delineation and spatial stability, likely attributable to its three-dimensional optics and improved instrument articulation, whereas the laparoscopic configuration achieved brighter overall illumination. These complementary features suggest that each system may offer distinct advantages depending on the surgical scenario, rather than implying superiority of one modality over the other.

A significant clinical challenge in the management of chylothorax lies in the anatomical variability of the canine thoracic duct, which may include collateral or plexiform configurations that complicate ligation [3,8,23,24]. Our findings underscore that ICG–NIR fluorescence facilitates the identification of these variants, emphasizing its value as an adjunct imaging technique rather than as a replacement for anatomical dissection skills. In clinical contexts, combining thoracic duct ligation (TDL) with cisterna chyli ablation or subtotal pericardiectomy achieves reported resolution rates of 85–95% [8,9]; thus, accurate intraoperative mapping remains essential to minimize postoperative persistence of effusion.

Minimally invasive thoracic surgery offers several clinical advantages. These include reduced postoperative pain, shorter recovery times, and improved respiratory outcomes compared to open thoracotomy [25]. However, recurrence remains a significant concern in idiopathic cases primarily due to incomplete ductal occlusion [4]. To address this, advanced imaging modalities, particularly the combination of preoperative computed tomography lymphangiography (CTLG) with intraoperative near-infrared fluorescence imaging, have been proposed to improve duct identification and surgical planning [6,18]. Our results support the technical feasibility of such multimodal approaches in veterinary thoracic surgery.

The Versius™ robotic platform facilitated thoracic duct dissection through the integration of modular robotic arm positioning, wristed instrumentation, and stable three-dimensional imaging capabilities. These functionalities enhanced dexterity and depth perception in confined spaces, facilitating precise and safe maneuvers that align with the advantages associated with robotic systems in intricate, minimally invasive surgical procedures [25]. Nevertheless, substantial limitations, including cost, accessibility, and a steep learning curve, persist as significant impediments to the widespread adoption of this technology within veterinary practice [19].

The limitations of this study include the use of cadaveric specimens lacking lymph propulsion, a small sample size, and potential variability between hemithoraces. Further in vivo studies are required to confirm reproducibility and translational value. The onset of fluorescence was delayed compared with reports in live dogs [6], probably due to absent lymphatic propulsion. Although fluorescence onset was delayed compared to live conditions, the cadaveric model allowed controlled evaluation of diffusion dynamics. No differences were observed between right and left thoracic approaches. However, the small and homogeneous sample size also limits extrapolation across breeds. Future clinical studies should evaluate recurrence rates, long-term outcomes, and cost-effectiveness of fluorescence-guided thoracoscopic strategies under clinical conditions.

Future research should focus on clinical validation of ICG–NIR-guided thoracic duct identification in live dogs, evaluating outcomes such as effusion resolution, recurrence, and perioperative morbidity. Research should also explore optimization of ICG protocols (dose, injection site, timing) to achieve consistent duct visualization across various clinical scenarios [17,26]. Additionally, continuous innovation is necessary to enhance the accessibility of robotic technology. This could include the development of more compact, veterinary-specific robotic systems or hybrid approaches that provide some of the advantages of robotics at a lower cost. As an interim step, certain surgeons are exploring handheld devices or 3D laparoscopes to bridge this gap [27,28,29].

In conclusion, this study provides the first descriptive assessment of a robot-assisted thoracoscopic approach for thoracic duct identification in dogs, integrating real-time indocyanine green (ICG) near-infrared (NIR) fluorescence within the Versius™ platform. While the use of ICG is already increasing in conventional thoracoscopic procedures, its application through a robotic system demonstrates the feasibility of combining fluorescence imaging with articulated instrumentation and three-dimensional vision in veterinary thoracic surgery. This integration enabled accurate duct localization and ergonomic dissection in the cadaveric model, highlighting its potential translational value for future clinical implementation. Further in vivo investigations are warranted to validate the reproducibility, safety, and long-term benefits of fluorescence-guided robotic thoracic duct surgery in canine patients. Overall, these findings provide a technical foundation for the clinical translation of fluorescence-guided robotic thoracic duct surgery in dogs.

## 5. Conclusions

This descriptive cadaveric study demonstrates the feasibility of thoracic duct identification using indocyanine green (ICG) near-infrared (NIR) fluorescence integrated into both video-assisted (VATS) and robot-assisted (RATS; Versius™) thoracoscopic platforms in dogs. Consistent duct visualization was achieved in all specimens, confirming that ICG-NIR fluorescence provides reliable anatomic delineation under minimally invasive conditions. While VATS generated higher overall signal intensity, the robotic platform provided enhanced contrast discrimination and image stability, facilitating accurate duct delineation.

These results do not aim to compare efficacy between the two modalities but rather to demonstrate the technical feasibility and imaging characteristics of fluorescence-guided thoracoscopic procedures performed with a robotic system. The integration of ICG fluorescence within a robotic platform represents an innovative approach for veterinary thoracic surgery and may provide a foundation for future translation into clinical management of canine chylothorax. Further in vivo investigations are warranted to assess reproducibility, clinical outcomes, and cost-effectiveness, as well as to refine dosing and imaging protocols for optimal lymphatic mapping.

## Figures and Tables

**Table 1:** Characteristics of canine cadaver specimens and fluorescence-guided thoracic duct visualization outcomes after indocyanine green (ICG) injection. All cadavers underwent both VATS and RATS sequentially. The data represent technical feasibility results without statistical inference. Operative times and visualization latency were recorded descriptively.

Cadaver ID	Body Weight (kg)	ICG Dose (mg)	Time to Visualization (min)	Notes
1	13.0	0.65	17	–
2	14.5	0.73	21	–
3	15.0	0.75	24	–
4	17.0	0.85	38	Required second ICG bolus

Abbreviations: ICG, indocyanine green.

**Table 2:** Descriptive optical imaging parameters for VATS and RATS. Values are presented as mean ± standard deviation (SD) and range. No statistical testing was applied, consistent with the exploratory study design.

Parameter	VATS (Mean ± SD, Range)	RATS (Mean ± SD, Range)
Time to TD visualization (min)	24.3 ± 9.7 (17–38)	24.3 ± 9.7 (17–38)
Total operative time (min)	35.0 ± 4.5 (29–41)	38.2 ± 5.1 (32–45)
Signal-to-noise ratio (SNR)	19.7 ± 10.9 (6.8–35.4)	15.2 ± 15.0 (4.5–33.2)
Contrast-to-noise ratio (CNR)	5.3 ± 4.4 (1.0–12.7)	11.7 ± 7.6 (3.2–21.5)
Contrast resolution (CR, %)	12.9 ± 9.8 (2.5–28.4)	40.1 ± 14.8 (18.9–61.5)

Abbreviations: TD, thoracic duct; SD, standard deviation.

## Data Availability

All data supporting the findings of this study are contained within the article. No additional datasets were generated or analyzed.

## Abbreviations

The following abbreviations are used in this manuscript:

TD	Thoracic Duct
TDL	Thoracic Duct Ligation
SD or StdDev	Standard Deviation
ICG	Indocyanine Green
NIR	Near-Infrared
VATS	Video-Assisted Thoracoscopic Surgery
RATS	Robot-Assisted Thoracoscopic Surgery
CNR	Contrast-to-Noise Ratio
SNR	Signal-to-Noise Ratio
CR	Contrast Resolution
CTLG	Computed Tomography Lymphangiography
CMR	Cambridge Medical Robotics.
UK	United Kingdom
ROI	Region of Interest
